# Thermosensitive black phosphorus hydrogel loaded with silver sulfadiazine promotes skin wound healing

**DOI:** 10.1186/s12951-023-02054-3

**Published:** 2023-09-15

**Authors:** Jie Zhou, Tianjiao Li, Meili Zhang, Bo Han, Tao Xia, Shuangshuang Ni, Zhiyong Liu, Zhenyang Chen, Xing Tian

**Affiliations:** 1 Key Laboratory of Xinjiang Phytomedicine Resource and Utilization, Ministry of Education, College of Pharmacy, Shihezi University, Shihezi, 832002 China; 2 Sinopharm Xinjiang Pharmaceutical Co. LTD, Urumqi, 830032 China; 3 College of Chemistry and Chemical Engineering, Shihezi University, Shihezi, 832003 China

**Keywords:** Black phosphorus nanosheet, Temperature sensitive hydrogel, Near-infrared light, Antibacterial, Wound healing

## Abstract

Wounds can lead to skin and soft tissue damage and their improper management may lead to the growth of pathogenic bacteria at the site of injury. Identifying better ways to promote wound healing is a major unmet need and biomedical materials with the ability to promote wound healing are urgently needed. Here, we report a thermosensitive black phosphorus hydrogel composed of black phosphorus nano-loaded drug silver sulfadiazine (SSD) and chitosan thermosensitive hydrogel for wound healing. The hydrogel has temperature-sensitive properties and enables the continuous release of SSD under near-infrared irradiation to achieve synergistic photothermal and antibacterial treatment. Additionally, it exerts antibacterial effects on *Staphylococcus aureus*. In a rat skin injury model, it promotes collagen deposition, boosts neovascularization, and suppresses inflammatory markers. In summary, the excellent thermosensitivity, biocompatibility, and wound-healing-promoting qualities of the reported thermosensitive hydrogel make it suitable as an ideal wound dressing in the clinic.

**Supplementary Information:**

The online version contains supplementary material available at 10.1186/s12951-023-02054-3.

## Introduction

The skin can sense environmental changes, defend the body from injury, and maintain physiological equilibrium [1]. As the biggest organ in the body, skin is crucial for preserving physiological homeostasis and shielding internal organs from the outside world. Cutaneous injuries, particularly those that cause chronic wounds, burns and skin wound infection, are a tremendous financial burden on healthcare systems across the world [2]. Several biomedical materials have been reported to prevent wound infection during the process of wound healing. However, traditional therapies such as antibiotics, often cause severe systemic side effects and contribute to antibiotic resistance [3]. Thus, safer and biocompatible strategies with fewer harmful side effects are urgently needed to enable wound healing and overcome antibiotic resistance associated with traditional therapeutic approaches.

Functional materials for biological applications have recently emerged due to the recent breakthroughs in the field of nanotechnology. Numerous disease-modifying therapeutic platforms based on nanomaterials have been developed so far [4, 5]. Among these, black phosphorus nanosheets (BPNSs) are a new class of materials with promising applications in electrochemistry, electronics, optoelectronics, environmental protection, biomedicine and other fields due to their wide bandgap, high anisotropy, wide optical absorption, high carrier mobility and a host of other characteristics [6]. Black phosphorus nanosheets mediate the combined photothermal and photodynamic therapy of cancers upon exposure to near-infrared light [7, 8]. Dextran hydrogels consisting of gelatin methacrylate (GelMA) and dextran oxide (oDex) also have black phosphorus (BP) nanosheets and zinc oxide nanoparticles added to them (ZnO NPs). The hydrogel displayed antibacterial effects due to the combination of its photothermal effects and zinc ions upon being exposed to 808 nm NIR laser [9]. Under NIR light irradiation, the Ag@BP nanocomposite continuously releases silver ions, exerts photothermal effects and rapidly demolishes the bacterial membrane, which limits bacterial growth [10]. Therefore, black phosphorus nanosheets have great prospects for medical applications.

An aqueous three-dimensional network called a hydrogel can be tailored to the physicochemical characteristics of human tissues, has high biocompatibility. Hydrogel can be made of natural or synthetic polymers. They are a novel class of non-irritating, non-toxic, and biocompatible polymers that have been extensively employed in the biomedical industry [11]. Due to their ability to retain water, hydrogels have garnered a lot of interest, offering a moist environment for wound healing [12]. Recent studies have focused particularly on thermosensitive hydrogels due to their unusual sol–gel transition features. At physiological temperature, these hydrogels display a phase shift [13–15]. Additionally, it enables the localized delivery of the loaded drugs, improving local drug concentration and avoiding the hazardous side effects brought on by systemic delivery. This is due to the fact that the hydrogels can be quickly gelled at the application site with simple temperature stimulation, avoiding the need for additional organic solvents, cross-linking substances, or external equipment [16]. In order to broaden their application potential, it is advantageous to create multifunctional hydrogels with superior mechanical qualities and light-to-heat conversion capabilities [17].

Black phosphorus nanosheets, as a new two-dimensional material, effectively reduce the oxidation of black phosphorus after the modification of polyethylene glycol by electrostatic adsorption, which not only increases the stability of black phosphorus nanosheets, but also improves its biocompatibility. Chitosan is a biocompatible material that is degraded by enzymes in vivo, and the degraded products are non-toxic oligosaccharides. In addition, chitosan enhances the permeability of drugs by modulating the tight junctions between epithelial cells, making chitosan a relatively popular research topic in the field of biomedicine. Chitosan/*β*-glycerophosphate has attracted a huge attention in the biomedical field due to its high biocompatibility, biodegradability, and sharp thermosensitive gelation. It is in a liquid state at room temperature, and upon minimally invasive application into the desired tissue, it forms a solid-like hydrogel due to elevated temperature. Hydroxypropyl cellulose has good adhesion and adheres to the tissue surface. As a clinical drug for the treatment of wound infection, silver sulfadiazine has broad-spectrum antibacterial activity and is widely used in new antibacterial wound dressings. Thus, thermosensitive black phosphorus hydrogel has great potential as a scaffold material in tissue engineering due to its good biocompatibility, high antibacterial property, and tissue adhesion.

In this study, a self-healing and temperature-sensitive black phosphorus hydrogel was designed. Black phosphorus nanosheets were modified with polyethylene glycol to increase their stability, loaded with the antibacterial drug silver sulfadiazine (SSD). Chitosan, β-glycerophosphate disodium salt and hydroxypropyl cellulose were prepared into thermosensitive hydrogel. The temperature sensitive hydrogel and black phosphorus loaded with drugs were physically mixed and showed good self-healing and bonding abilities. The thermosensitive black phosphorus hydrogel demonstrated high photothermal efficiency and antibacterial activities against *Escherichia coli* and *Staphylococcus aureus* when exposed to NIR light. Black phosphorus nanosheets with SSD showed synergistic antibacterial effects. Additionally, the hydrogel displayed good cytocompatibility against NIH3T3 cells in vitro and its ability to speed wound healing was demonstrated using a rat model of wound healing.

## Materials and methods

### Materials

Black phosphorus powder (BP) was purchased from Beijing Xianfeng Nano Co., LTD., chitosan (CS) and hydroxypropyl cellulose (HPC) were supplied by Yuan Ye Biological Co., LTD.. N-methylpyrrolidone (NMP) was purchased from Shanghai Maclin Biochemical Technology Co., LTD. and β-Disodium glycerophosphorate (β-GP) was purchased from Solarbio Co., LTD. Anhydrous ethanol was purchased from Tianjin Fengchuan Chemical Reagent Technology Co., LTD. and phosphate buffer solution (PBS) was purchased from Sangong Bioengineering (Shanghai) Co., LTD. Silver sulfadiazine was purchased from Max Company and acetic acid was purchased from Tianjin Fuyu Fine Chemical Co. LTD. Live-dead staining reagent was purchased from Beibo Biological Company and Cell Counting Kit-8 (CCK8) was purchased from MedChemExpress Company. Antibacterial tests was conducted using *Staphylococcus aureus* (*S. aureus*, ATCC 6538, Gram-positive).

### Preparation of black phosphorus nanosheets

Firstly, 4 mg black phosphorus powder was accurately weighed and fully ground in a mortar. The ground black phosphorus powder was dissolved in 2 mL N-methylpyrrolidone (NMP), placed in an ultrasonic cleaning machine for 6 h (ultrasonic 5 min, vortex 2 min), followed by centrifugation for 30 min (2000 rpm) at 4 °C to remove the larger sized particles and collect the supernatant, which was further centrifuged for 10 min (13,000 rpm, 4 °C). Then 1 mL PBS solution was added and the prepared black phosphorus nanosheet solution was wrapped in tin foil and stored at 4 °C.

### Polyethylene glycol modification of black phosphorus nanosheets

1.0 mL of 1 mg/mL black phosphorus nanosheet solution was taken and centrifuged at 13,000 rpm/min for 30 min, the supernatant was removed and dissolved it in 1 mL ultra-pure water. Then 10 mL PEG-NH2 was added, and the solution was vortexed for 3 min and subjected to ultrasound sonication for 10 min in the ice bath for 3 times. The solution was then placed in a shaker for 12 h (rotating speed 250 rpm, temperature 10 °C). After that, it was centrifuged at 13,000 rpm/min for 15 min to remove free PEG-NH2. After washing with ultra-pure water, the solution was again centrifuged at 13,000 rpm/min for 15 min and stored in PBS solution.

### Drug loading into black phosphorus nanosheets

SSD was dissolved in 0.1 mol/L dilute sulfuric acid and centrifuged at 13,000 rpm/min for 15 min to remove PBS. Then 10 mL of the solution (10 mg/mL) was added to the shaker for 24 h (rotating speed 250 rpm, temperature 10 °C). 10,000 rpm/min centrifugation to remove free SSD. The solution was again centrifuged at 10,000 rpm/min, the supernatant was removed and the precipitate containing BP-PEG-AgSD, was suspended in PBS and stored at 4 °C.

### Preparation of temperature-sensitive black phosphorus hydrogel

600 mg chitosan was added into 10 mL glacial acetic acid (0.15 mol) and allowed to dissolve. The solution was then autoclaved for 30 min to get chitosan solution. The β-sodium glycerophosphate solution was obtained by dissolving 1120 mg β-sodium glycerophosphate in 2 mL ultra-pure water and filtered through 0.22μm filter to remove bacteria. 2.50 g of hydroxypropyl cellulose was added into 25 mL ultra-pure water at 60 °C and stirred until completely dissolved, thereby generating the hydroxypropyl cellulose solution. The chitosan solution, β-sodium glycerophosphate solution, hydroxypropylocellulose solution and black phosphorus loading solution were prepared on an ice bath according to the volume ratio of 6:2:2:1 and were placed on a shaker until completely mixed, thereby forming temperature sensitive black phosphorus hydrogel.

### Characterization of BP@Gel

A drop of the 1 mg/mL BP and BP-PEG-AgSD solution were added into the copper net filled with carbon film and allowed to dry at room temperature after being ultrasonically dispersed in ethanol 100 times. By using a field emission transmission electron microscope (Hitachi HT7700) with an accelerated voltage of 200 kV, the microscopic morphology of the sample was examined. For observing the microstructure of the cross-section surface, the hydrogel sample was freeze-dried, sliced, and then coated with gold. The microstructure of the freeze-dried gel was examined using a scanning electron microscope (Hitachi SU8010). Malvern particle size analyzer was used to measure the particle size of BP, BP-PEG-NH2, and BP-PEG-AgSD solutions at a temperature of 25 °C. To determine the particle size of the sample, appropriate amount of solution was added to the potential cell under the above conditions. The freeze-dried CS/GP/HPC samples and purified CS , β-GP, and HPC powder were pressed with KBr dry powder. A Fourier Transform infrared spectrometer (Bruker Vertex 70v) was used to measure the infrared spectra of the samples. The resolution of the scanning wave number was 4 cm^−1^, and the range was 4000–400 cm^−1^. The thickness of the BP solution was analyzed using atomic force microscopy (MultiMode, Brock GMBH, Germany). The temperature-sensitive properties of hydrogels were observed by inverting the test tubes.

### In vitro stability evaluation of BP-PEG-AgSD

In order to study the stability of the BP-PEG-AgSD solution, different solvents were selected, namely PBS buffer and ultrapure water, and 1 mL of the solution was placed in a closed sample bottle, and the solution was kept on a constant temperature shaker at 37 °C for 7 days, and observe the color change at predetermined time intervals of 0, 3, and 7 days, while measuring its ultraviolet absorption wavelength.

### In vitro drug release

1 mL of CS/β-GP/HPC was added dropwise to a 10 mL sample bottle, and BP-PEG-AgSD was added dropwise to the gel at a volume ratio of 1:10. It was then gently shaken while adding, at 37 °C for 15 min until the hydrogel was formed. 1 mL of BP@Gel was added to the pretreated dialysis bag, added to 250 mL of PBS (pH7.4), and incubated at 37 °C on a constant temperature shaker with a rotation speed of 150 rpm/min. The hydrogel was then exposed to 808 nm laser irradiation for 10 min, divided into two groups, and the near-infrared intensity was 1 w/cm^2^. At 0 min, 20 min, 40 min, 1 h, 2 h, 4 h, 6 h, 8 h, 12 h, and 24 h, 1 mL of the solution outside the dialysis bag was collected, and an equal volume of release medium was added. UV spectrophotometer was used to detect the release of photothermally controlled drugs from BP@Gel. The absorbance was measured at 324 nm. The formula for calculating the drug release rate in vitro was as follows:

In vitro drug release rate % = (drug release amount / total drug amount) × 100%

### Adhesion and photothermal properties

Using fresh rat and pig skin tissue, the adhesion of hydrogels on the skin surface was studied. Firstly, fresh pig skin and mouse skin were cut into rectangles of equal size, and then 400 μL of gel and BP@Gel were applied to their surfaces respectively and placed in a glass petri dish. The petri dish with mouse skin and pig skin were placed at a constant temperature incubator at 37 °C, taken out after 10 min, and the gelation state of the hydrogel was observed. The mouse skin was stretched and the pig skin was placed vertically. The morphological changes of the hydrogel were observed, and pictures were taken.

Gel hydrogel, the water group, and the BP@Gel hydrogel group received a 10-min exposure to near-infrared (NIR) radiation at 808 nm and 1.0 W/cm^2^. Various laser powers were applied on the BP@Gel hydrogel. A thermography camera that uses infrared light was used to gauge the temperature of these samples at various time points. 808 nm laser was used to irradiate the hydrogel five times in order to increase its photothermal stability. With the use of an infrared camera, the temperature variations before and after NIR radiation were observed.

### Hemolysis assay

Red blood cells were collected from freshly anticoagulated mouse blood by centrifuging it for 15 min at 2000 rpm and 4 °C. The supernatant was then discarded. To create a red blood cell suspension for subsequent use, the red blood cells were diluted with PBS to a volume concentration of 2%. 500 μL of erythrocyte suspension should be added to a 1.5 mL centrifuge tube together with 500 μL of hydrogel extract. The tube should then be sealed and gently shaken before being incubated at 37 °C for an hour. Finally, 500 μL of 1% acetic acid should be added. Each group was paralleled three times, with Triton X-100 serving as the positive control and 500 μL of PBS serving as the negative control. A 96-well plate was filled with 100 μL of the supernatant after the mixture had been centrifuged at 2000 rpm and 4 °C for 15 min, and its absorbance at 540 nm was measured using a microplate reader. The hemolysis rate was determined using the following formula:

$${\text{Hemolysis rate }}\% \, = \,({\text{OD sample}}\, - \,{\text{OD negative}})/ \, ({\text{OD positivity}}\, - \,{\text{OD negative}}\, \times \,{1}00\%$$

The absorbance of the sample group is represented by OD sample, while the absorbance of the negative group is shown by OD negative. OD positive denotes the positive group's absorbance.

### Cytotoxicity and live-dead staining analysis

To obtain the hydrogel extracts, the sterilized hydrogel samples were placed in a 15 mL centrifuge tube filled with complete culture medium at a ratio of 0.1 g/mL mass/solution volume. The hydrogel samples were then incubated in a sterile incubator at 37 ± 0.5 °C for 24 h. Before applying the culture media to the seed plate, the old media from the culture bottle was removed, washed once with PBS, and 0.25% trypsin was added to dissociate the cells. After that the trypsin solution was removed and the cells were transferred to a new culture dish and suspended well in fresh complete media containing 10% fetal bovine serum. The cells were diluted with full media after being counted using the cell counting plates. 96-well plates were seeded with 0.5 × 10^4^ cells per well and cultured for 24 h at 37 °C with 5% CO_2_. The medium was then removed, and materials were added for 24 h, 48 h, and 72 h at various concentrations. The liquid was fully aspirated out and cleaned twice with PBS. Old media was removed and 100 μL of fresh DMEM medium with 10% fetal bovine serum was added to the wells, followed by the addition of 10 μL of CCK-8 solution to each well. The group containing culture medium and cells alone served as the control group. The absorbance of the wells were measured at 450 nm using an enzyme marker following a two-hour incubation in the cell culture incubator. The following formula was used to calculate the cell survival rate:

$${\text{Cell survival rate }}\left( \% \right)\, = \,\left( {{\text{A sample}} - {\text{A blank}}} \right) \, / \, \left( {{\text{A control}} - {\text{A blank}}} \right)\, \times \,{1}00\%$$

Only live cells were stained by calcein and AM. Since it is a nuclear staining dye, PI cannot cross the membranes of live cells. Instead, it enters the nucleus through the disorganized portion of the dead cell membrane, embeds in the DNA double helix, and produces red fluorescence (excitation:535 nm, emission:617 nm). Therefore, only dead cells are stained by PI. Live cells are hence green, whilst dead cells are red. The number of NIH3T3 cells was kept within 10 × 10^5^, and after being washed twice with PBS, the cells were stained for 15–20 min at 4 °C using a 200 μL staining solution. PBS was used to wash the cells, and laser confocal microscopy was used to visualize the cells (Leica TCS SP8).

### Antibacterial examination

Staphylococcus aureus Escherichia coli was utilized and cultivated overnight at 37 °C in Luria–Bertani (LB) medium at 150 rpm in Luria–Bertani (LB) media. The bacterial culture was centrifuged at 3000 rpm and reconstituted in PBS solution when the bacterial concentration reached 10^8^ CFU mL^−1^ (OD600 = 0.1) (0.01 M). After 2 h of incubation, the combination was exposed to an 808 nm laser with a power of 1 w/cm^2^ for 0, 3, 5, and 10 min. Dilute the resultant bacterial suspension serially, then add 100 μL of each sample to LB agar plates and incubate for 12 h at 37 °C. Each group's formative colony forming units (CFU) were photographed using a digital camera.

### Evaluation of wound healing in vivo

The Medica Ethics Committee of the First Affiliated Hospital of Shihezi University School of Medicine approved all the animal studies, which followed the country's regulations on the management of experimental animals [2022] Lun Audit (A2022-169-01). The male SD rats weighing around 200 g each, were purchased from the Experimental Animal Center of Xinjiang Medical University. After a week of acclimatization, all the rats were shaved. On the dorsal side of the SD rats, a full-layer wound model was created. In detail, after administering 10% chloral hydrate intraperitoneally to anesthetize the rats, two symmetrical full-layer defect wounds measuring 1 cm in diameter were made in the spine. There were four groups of 12 rats each: the Control group, the NIR group, the BP@Gel group, and the BP@Gel + NIR group. 1 ml of the hydrogel was injected into the wound in the BP@Gel group, and it was then exposed to 1W cm^2^ of near-infrared laser energy for 10 min. On days 0 through 14, a digital camera was used to take pictures of the wound. The wound area was determined using Image J software, and the wound healing rate was calculated as follows:

Wound healing rate (%) = (A0-AT)/ A0 × 100%, where A0 and At were the wound area on day 0 and day t, respectively.

### Analysis of cellular morphology

The wound edge skin and wound tissue of the 4 groups were collected on day 3, 7 and 14, respectively, and the organs, including heart, liver, spleen, lung and kidney were collected on day 14. The collected skin and organs were stored in the general tissue fixation solution (4% paraformaldehyde), and then the longitudinal Sects. (4 – 6 μm thick) were embedded with paraffin. The skin tissue sections of each group were subjected to HE staining, masson staining, immunohistochemistry and immunofluorescence, respectively. Immunohistochemistry was conducted to observe the expression of the pro-inflammatory factors, interleukin-6 (IL-6) and tumor necrosis factor-α (TNF-α). The expression levels of CD31 and CD68 were observed by immunofluorescence staining. Blood was collected on day 7 and 14 for serum biochemical tests.

### Statistical analysis

SPSS 27.0 software was used for the statistical analyses. Data were represented as mean ± standard deviation. To evaluate the variation between the two groups, students t-test was performed. To determine the statistical significance of more than two groups, one-way analysis of variance was employed. p values between (*) 0.05 and (**) 0.01 were regarded as statistically significant differences.

## Results and discussion

### Characterization of thermosensitive black phosphorus hydrogels

**Fig. 1:**
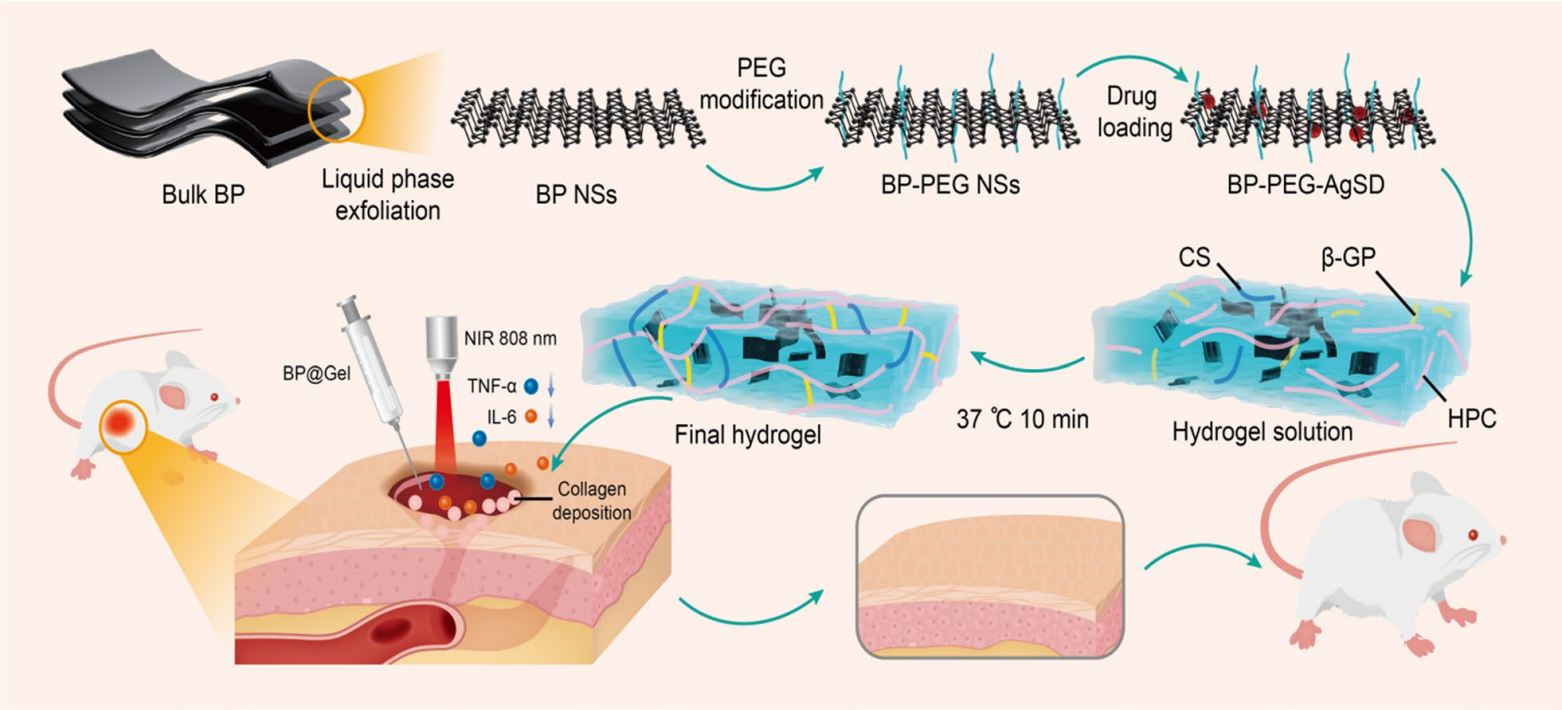
Schematic diagram of the mechanism of BP@Gel to promote skin wound healing.

**Fig. 2:**
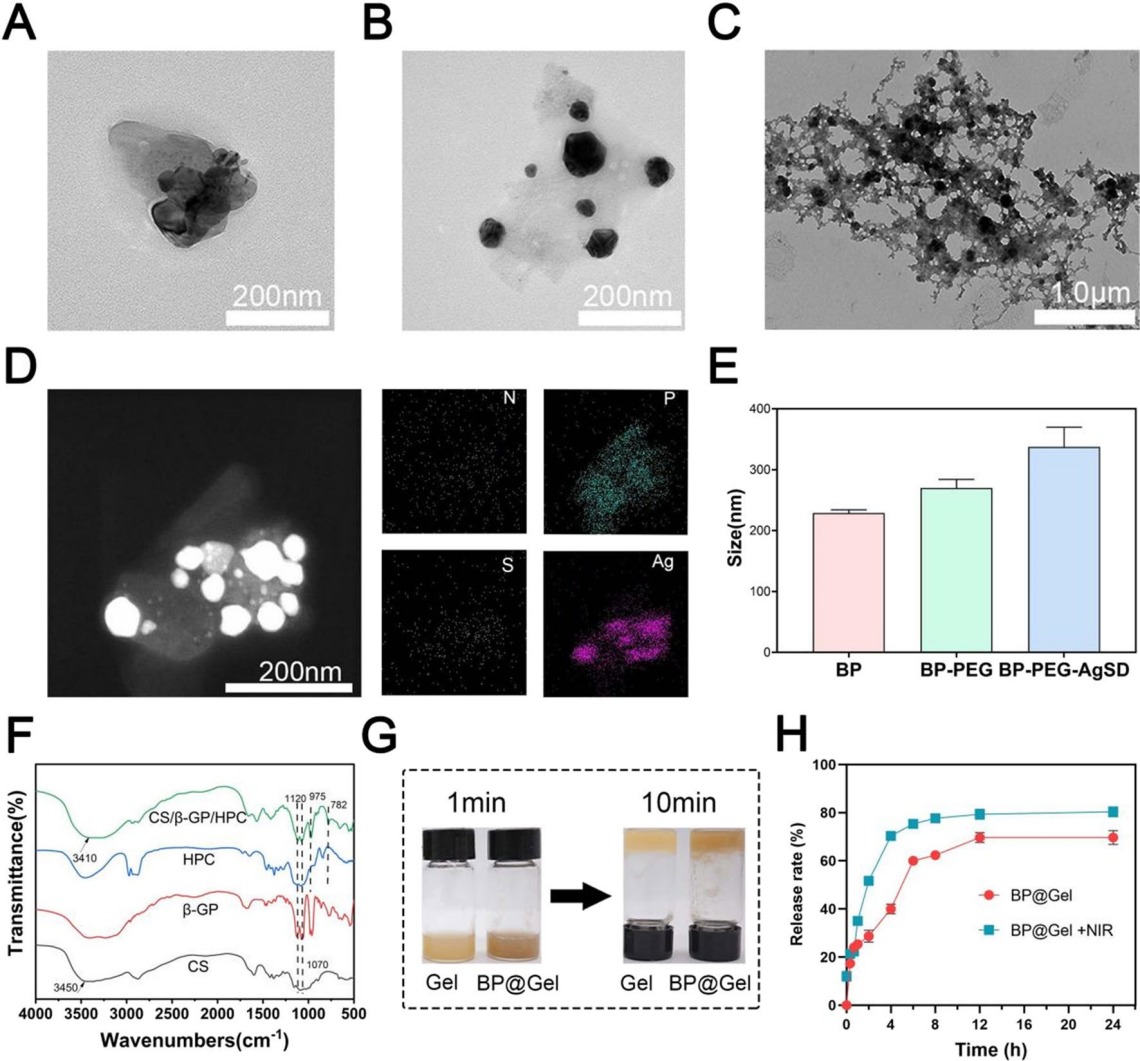
Characterization of BP@Gel. **A** TEM image of BP nanosheets. Scale bar: 200 nm. **B** TEM image of BP-PEG-AgSD. Scale bar: 200 nm. **C** SEM image of BP@Gel. Scale bar: 1.0 μm. **D** The scanning transmission map and elemental mapping map of BP-PEG-AgSD. Scale bar: 200 nm. **E** Particle size maps of BP, BP-PEG, and BP-PEG-AgSD nanosheets. **F** Infrared spectrum of CS/β-GP/HPC hydrogel. **G** Diagram of the sol–gel phase transition of Gel and BP@Gel. **H** In vitro drug release rate of BP@Gel

In this study, black phosphorus nanosheets were prepared by the liquid phase exfoliation method (Fig. 1) [18]. Transmission electron microscopy, atomic force microscopy, X-ray photoelectron spectroscopy, and Raman scattering were used to examine it. The black phosphorus nanosheets were found to have a homogeneous two-dimensional sheet structure, particle size of around 220 nm (Fig. 2A), and thickness of approximately 6 nm (Additional file 1: Fig. S3). The XPS peaks of P2p, P2s, C1s, and O1s of black phosphorus nanosheets were about 130 eV, 200 eV, 290 eV, and 510 eV, respectively (Additional file 1: Fig. S1). Raman scattering revealed three prominent peaks at 360, 438, and 465 cm (Additional file 1: Fig. S2). The above results showed that black phosphorus nanosheets were successfully prepared. The particle diameter of the black phosphorus nanosheet modified by polyethylene glycol was 266.7 nm and the particle diameter of the black phosphorus nanosheet after drug loading was 370.6 nm. The change in the particle size of black phosphorus nanosheets modified by PEG shows that the modification was relatively successful, and the particle size change after drug loading showed that SSD was loaded on black phosphorus nanosheets (Fig. 2E). Ag and S are the unique elements of the drug SSD, which can be seen from the mapping diagram of the transmission electron microscope. This further confirmed that SSD was successfully loaded (Fig. 2D). Elemental analysis by transmission electron microscopy showed that N, P, S, and Ag accounted for 3.64%, 6.46%, 0.13%, and 2.61% of the total atoms, respectively (Additional file 1: Fig. S4). We also investigated the stability of BP-PEG-AgSD solution, the color of BP-PEG-AgSD gradually faded on the 7th day, and the absorption spectra of day 0, 3rd day and 7th day showed that the absorption intensity decreased with time. At the same time, the storage of BP-PEG-AgSD in PBS increased its stability, making it a suitable for the storage of black phosphorus nanosheets (Additional file 1: Fig. S5). Thermosensitive black phosphorus hydrogel (BP@Gel hydrogel) was synthesized using black phosphorus nanosheet loaded with SSD (BP-PEG-AgSD) and chitosan thermosensitive hydrogel (Gel hydrogel), forming a three-dimensional network structure (Fig. 2C).

To verify the successful cross-linking of the hydrogels and that BP-PEG-AgSD had no effect on the chemical composition of the hydrogels, we performed FT-IR tests on the hydrogels. Infrared spectral features of CS, β-GP, HPC, and CS/β-GP/HPC hydrogels (Fig. 2F). The CS spectrum displayed the polymer's unique absorption peak. The stretching vibration of NH and OH in the CS structure correlated to the absorption peak at 3420 cm^−1^ [19]. This absorption peak blue-shifted from 3420 to 3410 cm^−1^ in the CS/β-GP hydrogel spectrum because the amino and hydroxyl groups in CS established hydrogen bonds with the hydroxyl groups in β-GP, lowering the generated by the electron cloud density of NH and OH [20]. Furthermore, because of the huge number of hydroxyl groups in β-GP, the absorption peaks of -OH in the hydrogel at 1120 cm^−1^ and 1070 cm^−1^ were sharper than those of CS. In the CS/β-GP hydrogel, additional absorption peaks were present at 975 cm^−1^ and 782 cm^−1^, respectively, which represented the symmetry of -GP stretching and bending vibration absorption peaks, in addition to the absorption peaks existing in CS. Based on the changes in these peaks, we concluded that there were a lot of hydrogen bonds in the CS/β-GP hydrogel. Since CS/β-GP/HPC was a simple physical mixture of HPC, no new absorption peaks appeared in the FTIR spectrum of HPC compared with CS/β-GP hydrogel, BP-PEG-AgSD was encapsulated in the hydrogel without changing the chemical structure (Additional file 1: Fig. S6). After inverting the test tubes, the gel hydrogel and BP@Gel hydrogel groups were placed in a constant temperature incubator at 37 °C. After 1 min, the solution was in a liquid state in the sample test tubes. However, after 10 min, they transformed into a gel, which indicated the temperature-sensitive properties of the above hydrogels (Fig. 2G). In general, the addition of BP-PEG-AgSD did not have a major impact on the gelation time of the hydrogel. Considering that the hydrogel would be injected into the skin wound and the physiological temperature is 37 °C, the hydrogel could seal the wound and the loaded drugs could be continuously released to promote wound healing.

The reason why black phosphorus is loaded with drugs is that on the honeycomb structure of black phosphorus nanosheets, after the phosphorus atom forms a bond with the other three phosphorus atoms, there are lone pairs of electrons, which are easily taken away by oxygen molecules, resulting in the oxidation of black phosphorus nanosheets. Therefore, the stability of black phosphorus nanosheets is increased by blocking its reaction with oxygen after surface modification with polyethylene glycol. Due to the presence of positively charged phosphate radicals on the surface of black phosphorus nanosheets, they can be combined with silver sulfadiazine through electrostatic adsorption. Chitosan forms intermolecular hydrogen bonds with sodium glycerophosphate and hydroxypropyl cellulose to form a three-dimensional network structure (Additional file 1: Fig. S7), and black phosphorus nanosheets loaded with silver sulfadiazine are wrapped in hydrogel. The above components constitute the temperature-sensitive black phosphorus hydrogel.

### Analysis of in vitro drug release

During the 24 h in vitro drug release experiment, it was found that the in vitro drug release rate of BP@Gel + NIR group was higher than that of the BP@Gel group. The drug release rate of BP@Gel + NIR group was 70.3 ± 1.5% at the 4 h, 75.7 ± 0.6% at 6 h, and 77.7 ± 1.5% at 8 h and tended to be stable; while the BP@Gel group reached 80.3 ± 2.1% at 24 h and tended to be stable. The overall drug release rate of the BP@Gel + NIR group was higher than that of the BP@Gel group (Fig. 2H).

### Adhesion and photothermal analysis

**Fig. 3:**
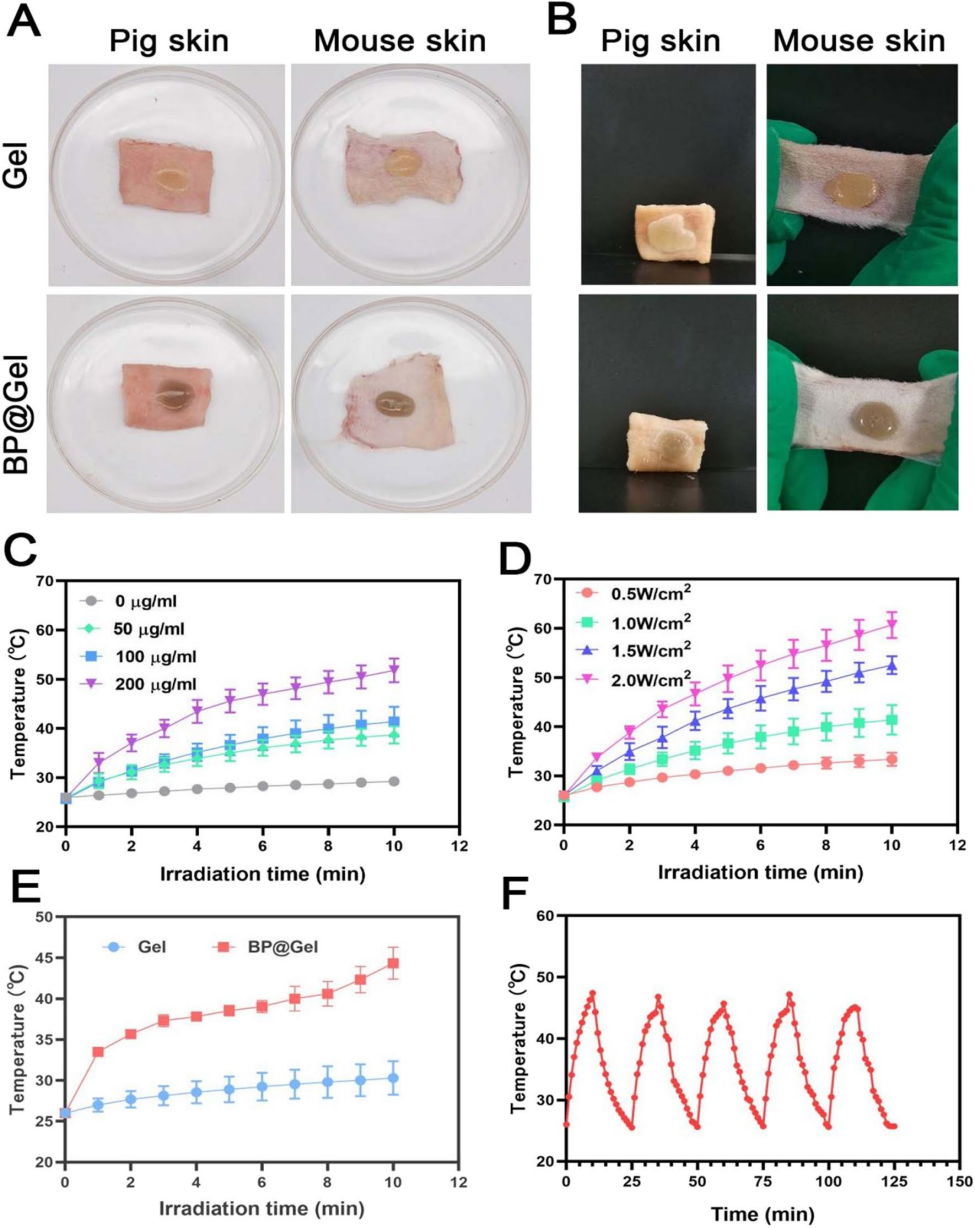
Adhesion and photothermal properties of BP@Gel. **A** Hydrogel spread on pig skin and mouse skin. **B** Vertical pig skin and stretched mouse skin.**C** Temperature variation curves of black phosphorus nanosheet dispersions with different concentrations under near-infrared (1W/cm^2^) irradiation. **D** 100 μg/mL black phosphorus nanosheet dispersion Temperature change of liquid under near-infrared irradiation with different powers. **E** Temperature change of hydrogel under near-infrared (1W/cm^2^) irradiation. **F** Five cycles of heating and cooling of BP@Gel

After 10 min, the Gel and BP@Gel formed a gel, which could be clearly seen in the petri dish, and the Gel and BP@Gel adhered to the mouse skin and pig skin. After stretching, the Gel and BP@Gel still adhered to the surface of the mouse skin and did not fall off. The pig skin was placed vertically, and the Gel and BP@Gel did not fall off, which indicated that BP@Gel had adhered well to the skin surface (Fig. 3A, B).

In order to further explore the photothermal effect and photothermal stability of black phosphorus nanosheets, the effect of NIR on different concentrations of black phosphorus nanosheets was evaluated in this experiment. As the concentration of black phosphorus nanosheets increased, the temperature increased, and the dispersion of 200 μg/mL black phosphorus nanosheets finally reached 63.6 °C at 1 W/cm^2^ (Fig. 3C). The concentration of the Gel group was 100 μg/mL Under different power irradiation, as the power increased, the temperature also increased (Fig. 3D). The Gel group and BP@Gel group were irradiated with NIR with a power density of 1.0 W/cm^2^ for 10 min, and the temperature changes were recorded by a thermocouple thermometer. After 10 min of irradiation, the final temperature of BP@Gel reached 45 °C. In contrast, almost no significant temperature change was observed in the Gel group, which indicated that the Gel itself had no photothermal effect. When BP-PEG-AgSD solution was added and irradiated with NIR with a power density of 1.0 W/cm^2^, the temperature increased with longer irradiation time (Fig. 3E).

Photothermal stability is one of the important factors to evaluate the effect of photothermal therapy. Poor photothermal stability limits the application of many biomaterials with photothermal properties in the biomedical field. In order to evaluate the photothermal stability of BP@Gel, five consecutive NIR light cycles were performed using the switching mode. The temperature of BP@Gel increased rapidly within 10 min, and after turning off the laser for 15 min, the BP@Gel cooled down and reached the initial temperature. In five consecutive NIR (irradiation-cooling) cycles, it was found that the maximum photothermal conversion temperature of BP@Gel did not change substantially, which suggested that BP@Gel had good photothermal stability. Based on the excellent photothermal effect and photothermal stability of BP@Gel, BP@Gel may serve as a promising candidate material for PTT (Fig. 3F).

### Evaluation of hemocompatibility and cytocompatibility in vitro

**Fig. 4:**
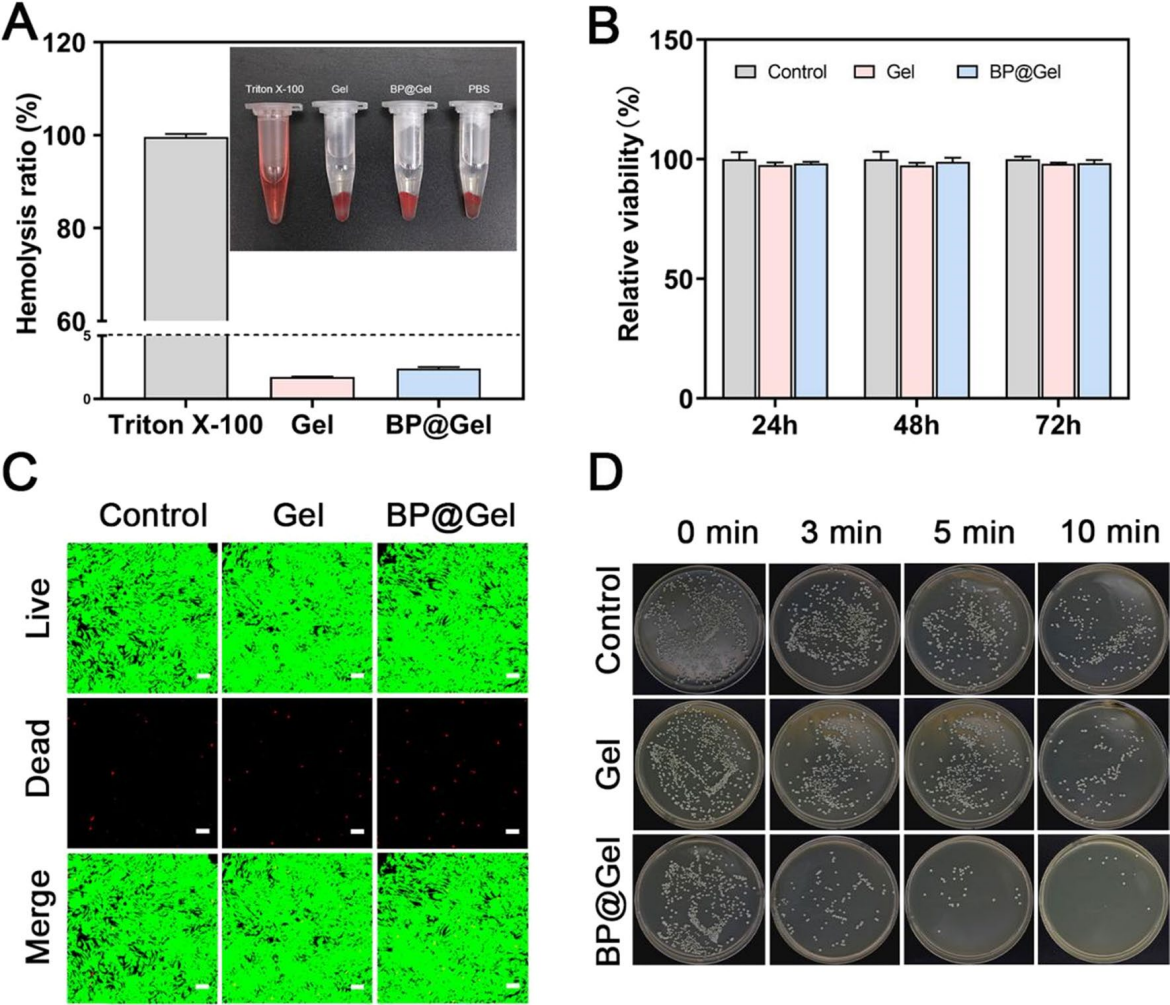
In vitro experiment of BP@Gel.**A** Hemolysis rate of hydrogel and control group. **B** In vitro cell viability assay of the hydrogel. **C** In vitro NIH3T3 cell live-dead fluorescent staining of the hydrogel. **D** Near-infrared (1W/cm^2^) images of different groups of Staphylococcus aureus

Nanocomposite hydrogels must be biocompatible before they can be used in the clinic. Hemolysis assay is used to assess the cytotoxicity of medicinal agents and is also used to characterize blood contact materials. According to the American Society for Testing and Material Designation (ASTMF-756-00), a hemolysis rate (HR) greater than 5% is considered as severe hemolysis, while a hemolysis rate less than 2% and between 2 and 5% is deemed non-hemolysis, and mild hemolysis, respectively [21]. The hemocompatibility of BP@Gel hydrogel was quantified using a hemolysis test, with Triton X-100 as a positive control and PBS as a negative control [22]. The positive control group's supernatant was bright red, with no sediment at the bottom, indicating that the cells were destroyed and severe hemolysis had occurred, whereas the negative control group, Gel group, and BP@Gel hydrogel group's supernatants were clear and transparent, with a large pellet of red blood cells at the bottom. The HR of the hydrogels in the Gel hydrogel group and the BP@Gel hydrogel group were both less than 3% (Fig. 4A), showing that the hydrogels were hemocompatible.

NIH3T3 cells and hydrogel extracts were used as in vitro models for the viability and live/dead fluorescence labeling experiments. First, NIH3T3 cells were co-cultured with various doses of hydrogel extracts for 24 h, 48 h, and 72 h, and the OD value was measured using the CCK8 assay. The cell viability of each group was higher than 85% at 24 h, 48 h, and 72 h, and the cell viability at different concentrations of hydrogel extracts was greater than 85% at all the tested time points (Fig. 4B, Additional file 1: Fig. S8). For the live/dead cell staining, the cells were co-cultured with the hydrogel extract for 72 h before being labeled with the live/dead reagents and visualized using a confocal microscope (Leica TCS SP8) (Fig. 4C). The results revealed that the Gel hydrogel group had a small number of dead cells (red), with most of them being live cells (green). The morphology of live cells in the BP@Gel hydrogel group was changed from irregular polygons or triangles to circles after 72 h due to the addition of black phosphorus nanosheets, while the number of dead cells increased. In general, the viability of NIH-3T3 cells in each experimental group was high, indicating good cytocompatibility of the hydrogel.

### Antimicrobial properties of the hydrogels

In this study, a novel BP@Gel hydrogel-based wound dressing with antibacterial activity was prepared. Using the plate spread method, *Staphylococcus aureus* (Gram-positive bacteria) was selected as a representative model of bacteria. The bacterial viability of *Staphylococcus aureus* was significantly reduced after incubation with BP@Gel hydrogel under NIR light irradiation. The Gel hydrogel group alone did not show this antibacterial effect on *Staphylococcus aureus*. Based on the results of the Control group, when NIR light was irradiated for 10 min, it could kill some bacteria. At the same time, the Gel group had an antibacterial effect at 10 min. It could be that chitosan itself possessed antibacterial properties and could inhibit the growth of bacteria. When NIR radiation was applied for 10 min, the BP@Gel group had a significant antibacterial effect compared to the Control group and the Gel group. At the same time, the antibacterial effect of the BP@Gel group decreased without NIR irradiation. The above results showed that the combined application of NIR irradiation and hydrogel could achieve synergistic antibacterial effects and that the combination of BP@Gel and NIR irradiation was enough to kill most of the bacteria (Fig. 4D).

### Evaluation of wound healing in in vivo mouse models of skin wound

Hydrogels are commonly used as skin wound dressings to keep wounds wet and promote tissue healing [23, 24]. Wound healing is a dynamic, well-controlled process that involves partially overlapping stages, including hemostasis, inflammation, proliferation, and remodeling [25, 26]. The antibacterial agent loaded onto the black phosphorus nanosheets in this study was SSD. SSD is an FDA-approved topical antibiotic that has been reported to be effective against Gram-negative and Gram-positive bacteria [27]. In addition, SSD possesses wound-healing effect [28–30].

**Fig. 5:**
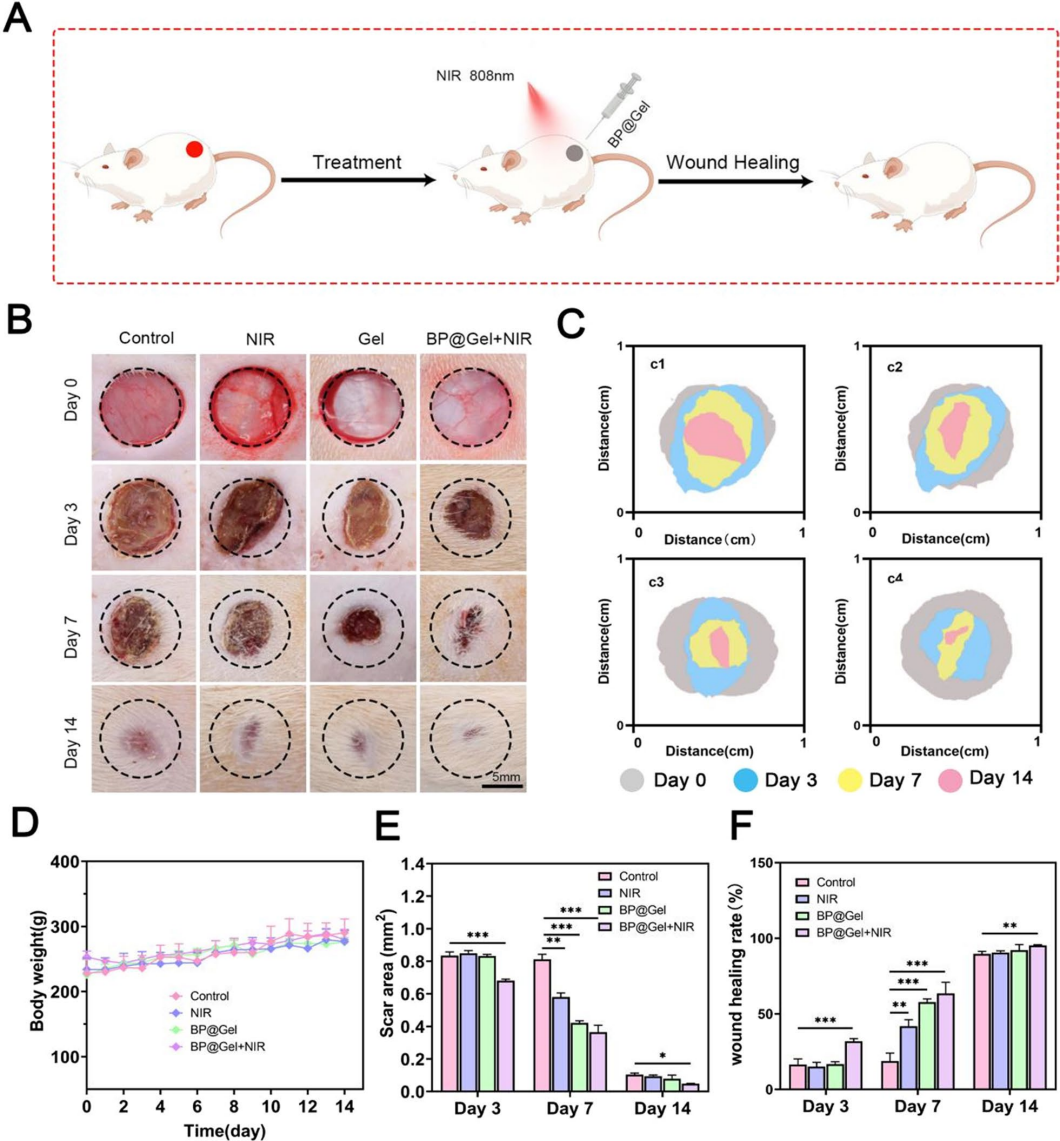
Effects of thermosensitive black phosphorus hydrogel on wound healing in vivo. **A** Schematic illustration of hydrogel promoting wound healing. **B** Wound images of different groups after the wound area was treated on days 0, 3, 7, and 14 after surgical resection. Scale bar: 5 mm. **C** Schematic diagram of wound healing simulated by image software in Control group (c1), NIR group (c2), BP@Gel group (c3) and BP@Gel + NIR group (c4). **D** Graph of body weight change in rats during wound healing. **E** Wound area map. **F** Graph of wound healing rate. (*P < 0.05, **P < 0.01, ***P < 0.001)

The rat skin full thickness wound model was used to evaluate the in vivo wound healing properties of thermosensitive black phosphorus hydrogel as a potential wound dressing. A circular full-thickness skin defect wound with a diameter of 1 cm was generated on the back of SD rats. BP@Gel hydrogel was then injected into the wound and irradiated with 808 nm near-infrared light for 10 min to promote wound healing (Fig. 5A). After treating the wounds in different groups, wound healing was evaluated (Fig. 5B, C). On the third day, the wound area of the BP@Gel hydrogel group was found to be significantly smaller than that of the other groups. On day 7, the control group had some exudate along with redness and swelling, while the wound in the BP@Gel hydrogel group had almost no swelling or redness. This indicated that BP@Gel hydrogel was able to reduce wound inflammation, which was potentially mediated by the sustained release of black phosphorus nanosheets and SSD from the hydrogels. The separate Gel group containing chitosan possessed wound healing ability, enhanced wound skin metabolism, covered and isolated ulcer wounds, inhibited bacterial growth and protected wounds, and inhibited scar formation [31, 32]. We showed that the BP@Gel hydrogel released black phosphorus nanosheets and SSD simultaneously, which was more effective than the Gel hydrogel group alone, and the Gel hydrogel group containing chitosan also promoted wound healing. On day 14, the wounds in the different groups were basically healed, but those in the BP@Gel hydrogel group healed better (Fig. 5E, F). From day 0 to day 14, the body weight of the rats in different groups gradually increased, indicating that the treatments in different groups did not change the body weight of the rats (Fig. 5D). Overall, the BP@Gel hydrogel group had the smallest scar area and the best skin regeneration ability compared with the other experimental groups.

### Histological assessment of the regenerated tissue

**Fig. 6:**
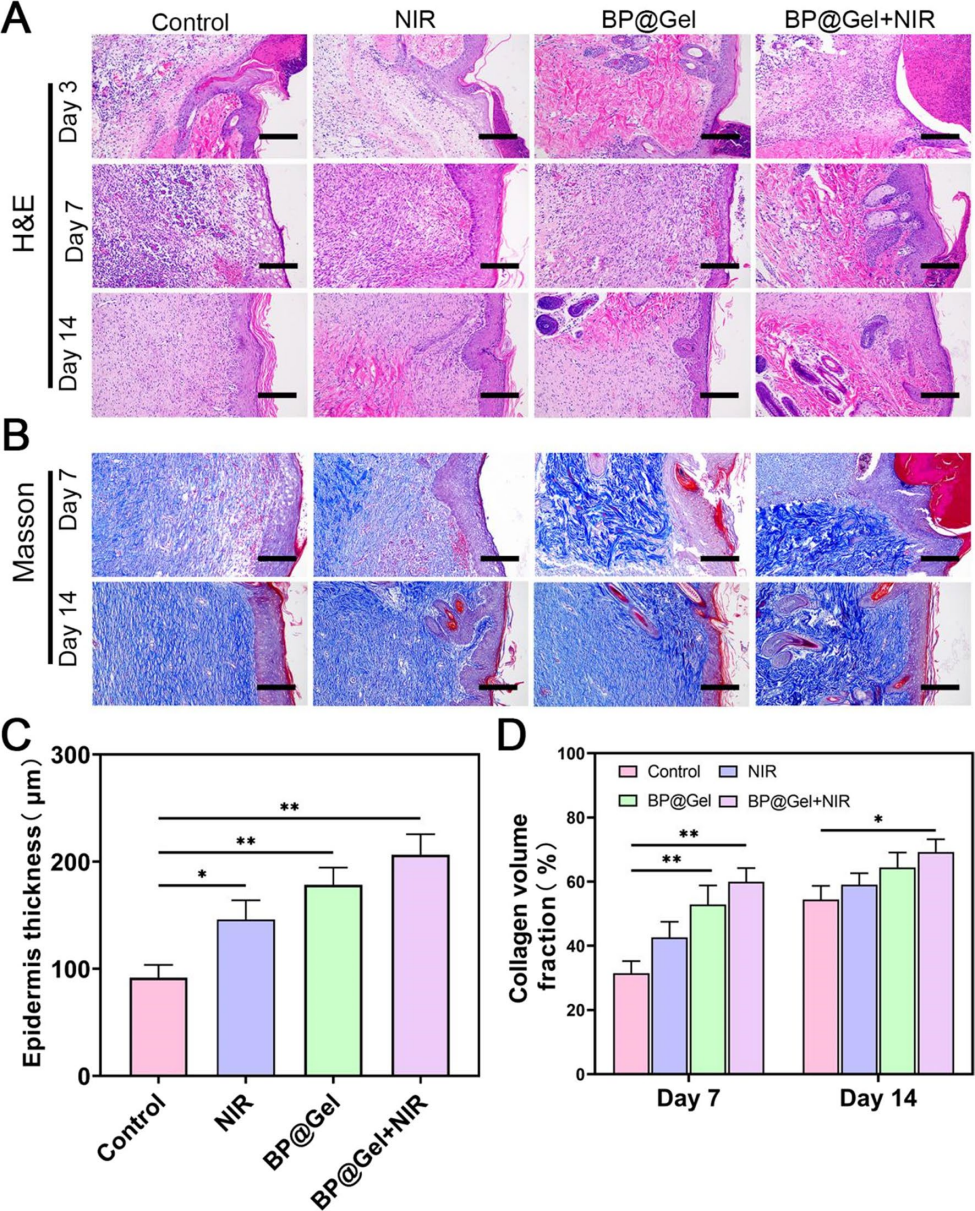
Histological analysis of skin wounds in different groups on day 3, day 7 and day 14. **A** HE staining and **B** Masson staining results of skin tissue. Scale bar: 100 μm. **C** Epidermal thickness map at day 7. **D** Masson staining quantitative analysis of collagen fiber content (%) in different groups. (*P < 0.05, **P < 0.01)

Histological evaluation elucidates the tissue regeneration process and reveals the therapeutic effect of the hydrogel on skin wounds [33, 34]. To assess wound healing, HE staining was conducted on days 3, 7, and 14. On day 7, the dermis of the NIR group consisted of a substantial number of inflammatory cells penetrating the granulation tissue, but the wounds in the Gel hydrogel group and the BP@Gel hydrogel group contained fewer inflammatory cells and established an epithelial layer. This was potentially due to the presence of chitosan in the Gel hydrogel group, which promoted wound healing, and at the same time, SSD loaded on the black phosphorus nanosheets promoted antibacterial effects and synergized with photothermal effects from the nanosheets. It was previously shown that black phosphorus nanosheets coated with silver nanoparticles were extremely efficient at inducing antibacterial effects in vivo [35]. The skin tissue structure of the BP@Gel hydrogel group was normal on the 14th day, as were the morphology and structure of the skin appendages, and there was no inflammatory cell infiltration in the tissue (Fig. 6A). Furthermore, the thickness of the newly created epidermis increased to varying degrees in each group on day 7, with the thickness of the Control group, NIR group, Gel group, and BP@Gel + NIR group being 91.76, 146.03, 178.27, and 206.47 μm, respectively (Fig. 6C). The fundamental structure of the epidermis and dermis were present on the wound surface of the Control group on day 14, but the dermis was still in the process of repair, the inflammatory reaction was mild and no skin appendages were detected. The skin structure was slightly aberrant in the NIR group, and some collagen fibers in the dermis were absent. In contrast, the connective tissue and epithelial cells in the Gel hydrogel and BP@Gel hydrogel groups appeared normal with more fibroblasts, new blood vessels, hair follicles, the collagen fibers in the dermis were neatly arranged, and the morphology and structure of the skin appendages were also normal. There was no major inflammatory response in the two groups, indicating that the two hydrogels possessed good wound healing properties. On day 14, the BP@Gel hydrogel group showed the best wound repair result, with more fibroblasts and hair follicles than in the other three groups.

Masson staining was performed to evaluate collagen fibers during wound healing [36]. On days 7 and 14, all groups showed collagen deposition. The control group showed less collagen deposition on the wound surface than the other groups. The collagen fibers were denser, thicker and better ordered in the BP@Gel hydrogel group, which promoted wound healing with the highest collagen density and the most orderly fiber structure, akin to natural skin (Fig. 6B). The collagen volume percent was greatest in the BP@Gel hydrogel group (Fig. 6D). The above results demonstrated that BP@Gel hydrogel greatly increased collagen deposition during wound healing.

### IL-6, TNF-α, CD31, and CD68 expression during wound healing

**Fig. 7:**
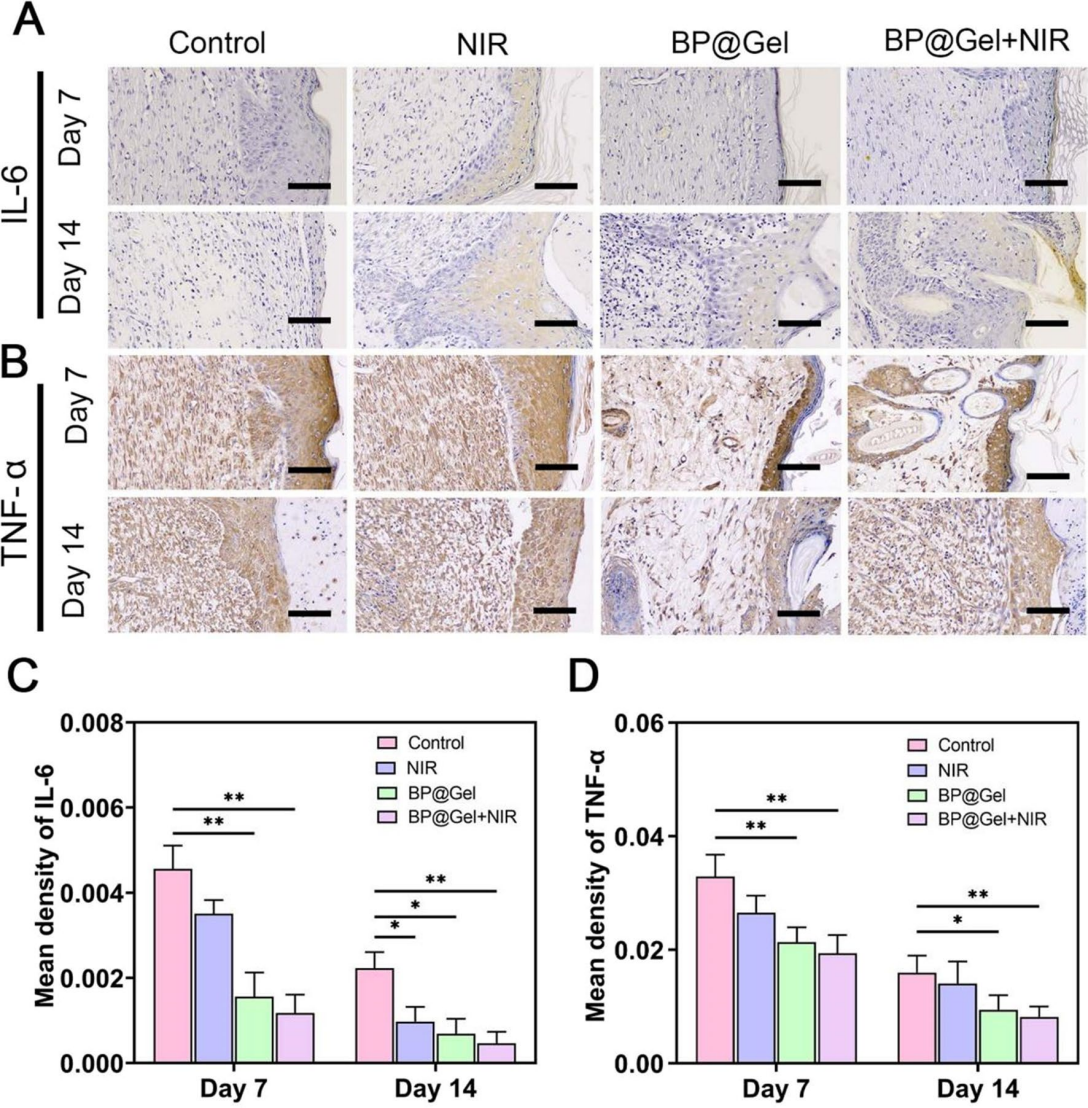
Immunohistochemical analysis of inflammatory factors. **A** Pictures of immunohistochemical staining of IL-6 and **B** TNF-α in skin tissues on day 7 and day 14. Scale bar: 100 µm. **C** B) IL-6 content and **D** TNF-α content in regeneration on days 7 and 14. (*P < 0.05, **P < 0.01)

To further investigate the mechanism of BP@Gel hydrogel mediated wound healing, the factors influencing the wound healing process were examined, particularly the expression of inflammatory angiogenic factors. IL-6 and TNF-α are typical pro-inflammatory cytokines involved in the early stage of wound healing when the skin wound is in the inflammatory phase, and they are essential markers of inflammatory response [37–40]. There was a very low expression of IL-6 and TNF-α in the BP@Gel hydrogel group compared with the other groups (Fig. 7A–D), which was similar to the results from HE staining (Fig. 6A). This indicated that BP@Gel hydrogel had good anti-inflammatory properties.

**Fig. 8:**
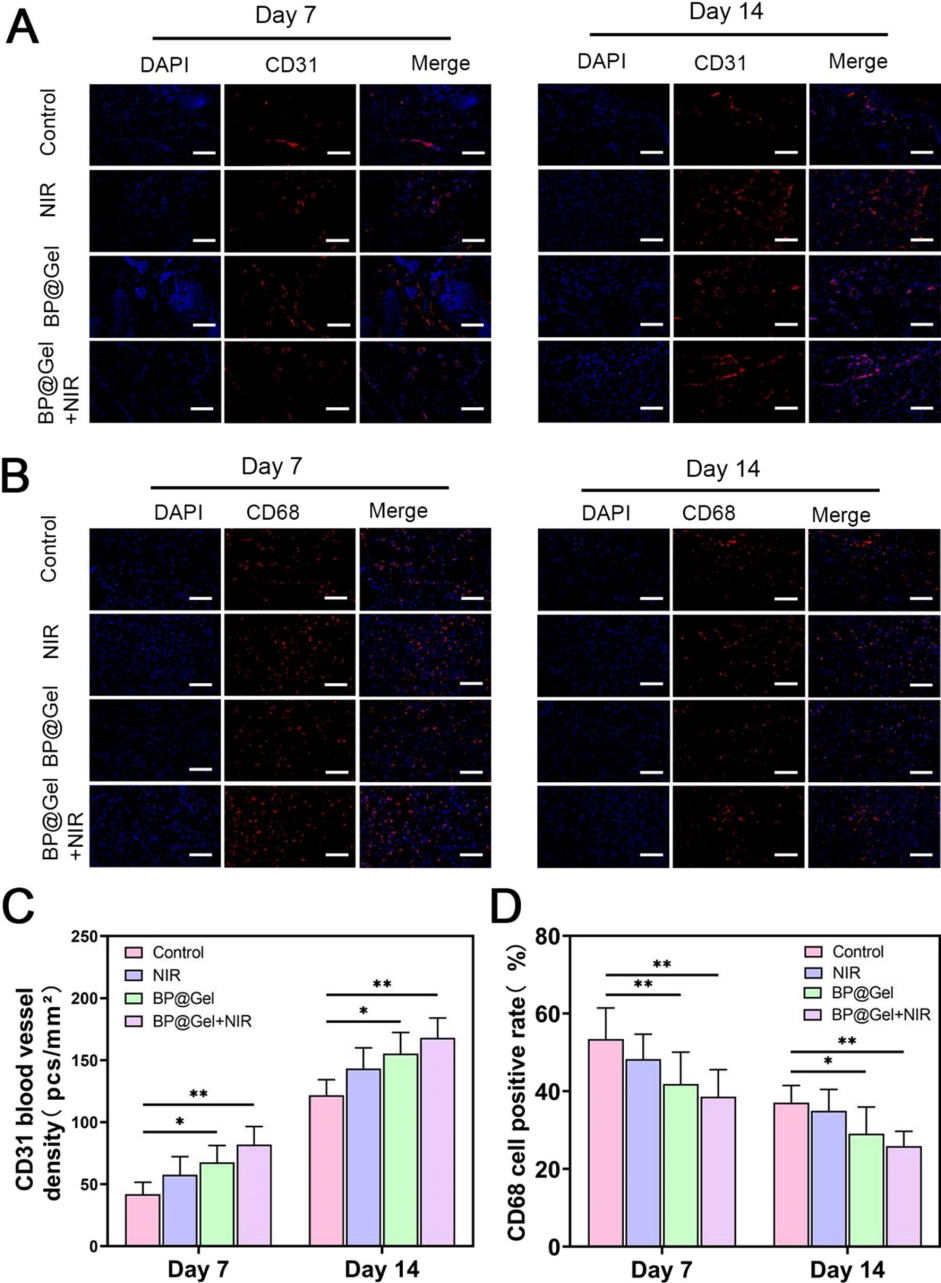
Immunofluorescence analysis of neovascularization and macrophages in wounds and HE staining of organs. **A** Immunofluorescence images of CD31 and **B** CD68 on days 7 and 14. Scale bar: 100 µm. **C** Density of CD31 neovascularization. **D** CD68 positive cell rate. (*P < 0.05, **P < 0.01)

In addition, the expression of cytokines associated with inflammation (CD68) and angiogenesis (CD31) were evaluated during skin regeneration [41, 42]. Wound neovascularization was examined by immunofluorescent labeling with CD31 after 7 and 14 days of therapy. The wounds treated by the Gel group and the BP@Gel hydrogel group showed higher CD31 expression, and the CD31 blood vessel density increased dramatically (Fig. 8C), demonstrating that the BP@Gel hydrogel promoted wound closure and induced angiogenesis. Similarly, immunofluorescence staining for CD68 was lower in the BP@Gel hydrogel group compared to the other groups (Fig. 8D). In general, treatment with BP@Gel hydrogel induced angiogenesis and inhibited inflammatory cytokine expression during the wound healing process.

### In vivo biocompatibility of thermosensitive black phosphorus hydrogel

**Fig. 9:**
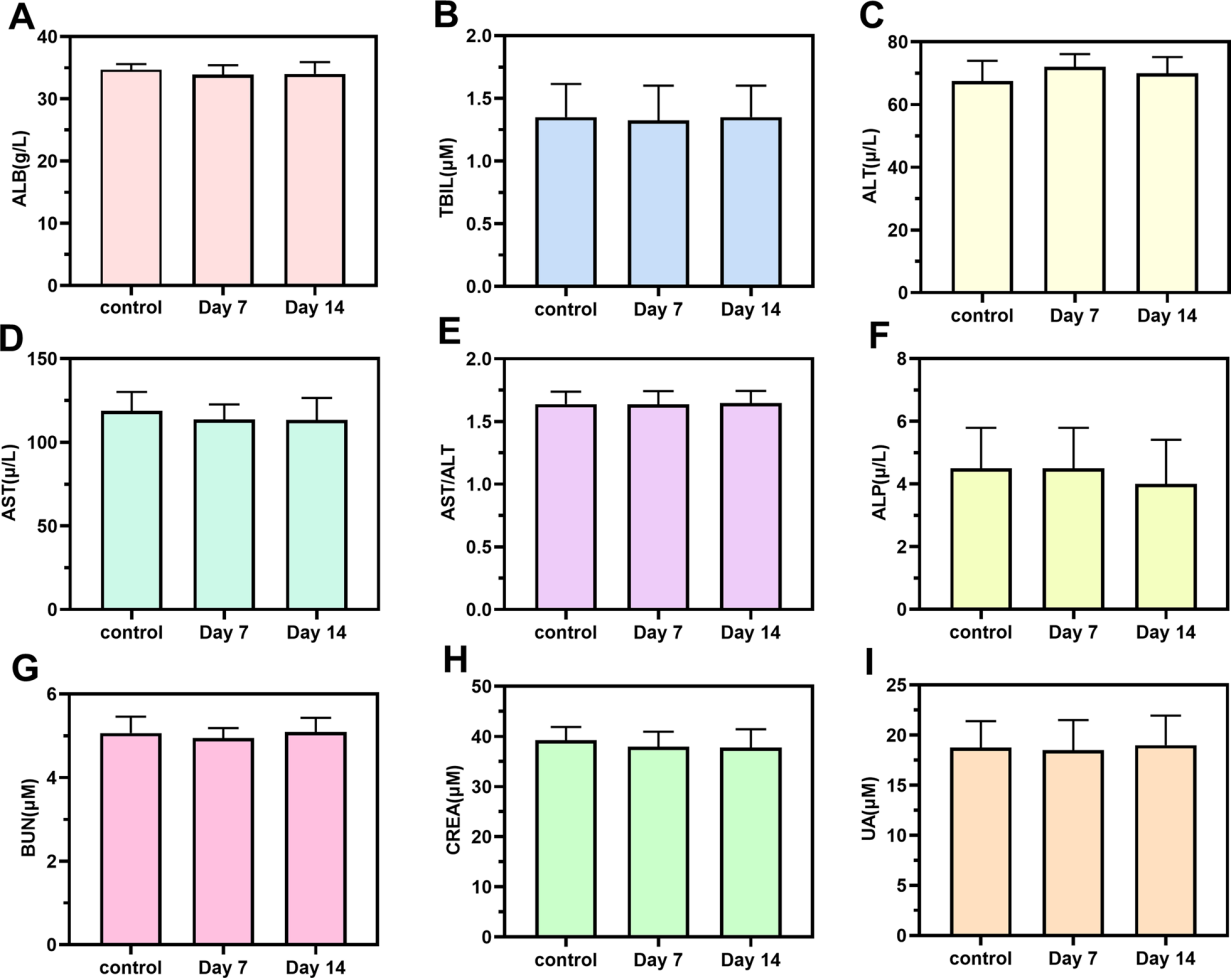
Serum biochemical indicators of BP@Gel hydrogel on day 7 and day 14. **A** ALB. **B** TBIL. **C** ALT. **D** AST. **E** AST/ALT. **F** ALP. **G** BUN. **H** CREA. **I** UA

Biosafety is an important criterion for the application of nanomaterials in biomedicine. The in vivo biocompatibility of temperature-sensitive black phosphorus hydrogel was comprehensively analyzed to ensure their in vivo safety. On days 7 and 14 following dosing, blood biochemical markers were examined. As shown in Fig. 9A–I, blood biochemical analysis revealed that albumin (ALB), alanine transferase (ALT), aspartate transferase (AST), aspartate/glucoside (AST/ALT), alkaline phosphatase (ALP), blood urea nitrogen (BUN), creatinine (CREA), uric acid (UN), and other major blood parameters were not significantly different between the control group and the experimental group. Simultaneously, HE staining was conducted for histological evaluation of the primary organs of rats, including the heart, liver, kidney, spleen, lung and brain, in order to further analyze the toxicity of BP@Gel to near-infrared-irradiated rats. Histological examination revealed no differences between the different groups, indicating the in vivo safety of BP@Gel (Additional file 1: Fig. S9). The BP@Gel discharged at the wound site did not promote systemic toxicity, which indicated that the thermosensitive black phosphorus hydrogel had high biocompatibility in vivo and was suitable as a new wound dressing to promote wound healing.

## Conclusion

This study reports the development of a thermosensitive black phosphorus hydrogel that could aid in wound healing. The results demonstrated that the hydrogel possessed good temperature-sensitive characteristics. To achieve synergistic therapy with photothermal and antibacterial agents were loaded on to the black phosphorus nanosheets, which augmented the photothermal effects. Then SSD was loaded and the thermosensitive hydrogels showed good biocompatibility. Hydrogels offer a moist microenvironment for skin wounds to heal faster, absorb wound exudate, and shield wounds from infection. The hydrogel's ability to promote wound healing was improved by the sustained release of the loaded drugs from the black phosphorus nanosheets. Thus, a functional hydrogel was designed to promote wound regeneration in patients. The hydrogel synergized antibacterial and photothermal properties to promote wound healing, which may be applied to burns and scalds in the future, offering a promising potential for its clinical application.

### Supplementary Information

**Additional file 1: Figure S1.** XPS pattern of black phosphorus nanosheets. **Figure S2.** Raman scattering pattern of black phosphorus nanosheets. **Figure S3.** AFM image of black phosphorus nanosheets. **Figure S4.** Mapping diagram of BP-PEG-AgSD. (A) TEM image of BP-PEG-AgSD. (B) Quantitative analysis chart of each element. (C) EDX image of TEM. **Figure S5.** Stability performance of BP-PEG-AgSD solution. (A) Stability pictures of different time periods (B) and (C) Absorption spectra of BP-PEG-AgSD solution in PBS and water at day 0, day 3 and day 7. **Figure S6.** Infrared spectra of AgSD, BP-NSs, BP-PEG-AgSD and BP@Gel. **Figure S7.** Reaction formula of chitosan, sodium *β*-glycerophosphate and hydroxypropyl cellulose. **Figure S8.** NIH3T3 cytotoxicity graph of Gel hydrogel group and BP@Gel hydrogel group. (A-C) Gel hydrogel groups at 24 h, 48 h and 72 h. (D-F) BP@Gel hydrogel group at 24 h, 48 h and 72 h. **Figure S9.** HE staining images of organs in Control group and BP@Gel hydrogel group. Scale bar: 100 nm.

## Data Availability

The data that support the findings ofthis study are available from thecorresponding author upon reasonablerequest.
