# Supplementary material for: Thermosensitive black phosphorus hydrogel loaded with silver sulfadiazine promotes skin wound healing
Source: J Nanobiotechnology. 2023 Sep 15;21:330. doi: 10.1186/s12951-023-02054-3 (PMC10503145; doi:10.1186/s12951-023-02054-3)
Supplement: Supplementary file 1 — Additional file 1: Figure S1. XPS pattern of black phosphorus nanosheets. Figure S2. Raman scattering pattern of black phosphorus nanosheets. Figure S3. AFM image of black phosphorus nanosheets. Figure S4. Mapping diagram of BP-PEG-AgSD. (A) TEM image of BP-PEG-AgSD. (B) Quantitative analysis chart of each element. (C) EDX image of TEM. Figure S5. Stability performance of BP-PEG-AgSD solution. (A) Stability pictures of different time periods (B) and (C) Absorption spectra of BP-PEG-AgSD solution in PBS and water at day 0, day 3 and day 7. Figure S6. Infrared spectra of AgSD, BP-NSs, BP-PEG-AgSD and BP@Gel. Figure S7. Reaction formula of chitosan, sodium β-glycerophosphate and hydroxypropyl cellulose. Figure S8. NIH3T3 cytotoxicity graph of Gel hydrogel group and BP@Gel hydrogel group. (A-C) Gel hydrogel groups at 24 h, 48 h and 72 h. (D-F) BP@Gel hydrogel group at 24 h, 48 h and 72 h. Figure S9. HE staining images of organs in Control group and BP@Gel hydrogel group. Scale bar: 100 nm. [file 12951_2023_2054_MOESM1_ESM.docx]

**Additional file**

Thermosensitive black phosphorus hydrogel loaded with silver sulfadiazine promotes skin wound healing

Jie Zhou^a^, Tianjiao Li^a^, Meili Zhang^a^，Bo Han^a^,Tao Xia^b^, Shuangshuang Ni^b^, Zhiyong Liu^c,^*****, Zhenyang Chen^b,^******, Xing Tian^a,b,^*******

**^a^Key Laboratory of Xinjiang Phytomedicine Resource and Utilization, Ministry of**

**Education, College of Pharmacy, Shihezi University,Shihezi, 832002, China**

**^b^Sinopharm Xinjiang Pharmaceutical Co. LTD, Urumqi,830032, China**

**^c^College of Chemistry and Chemical Engineering, Shihezi University, Shihezi, 832003, China**

***Corresponding author**

****Corresponding author**

*****Corresponding author**

**E-mail addresses:lzyongclin@sina.com (Z. Liu),8809358@qq.com (Z. Chen), tianxingdeyoujian@163.com (X. Tian)**


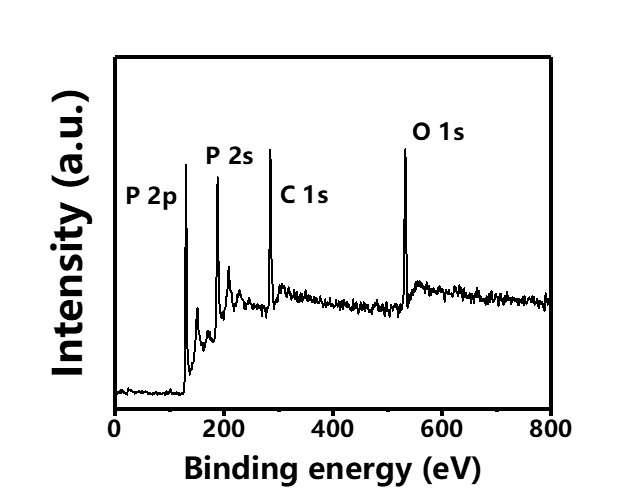

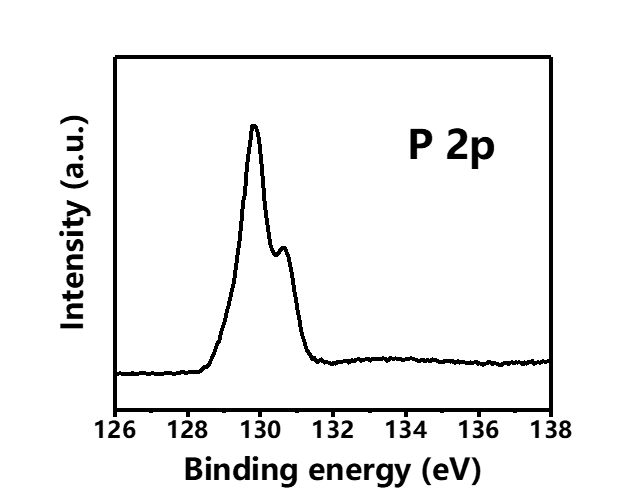


Figure S1. XPS pattern of black phosphorus nanosheets.


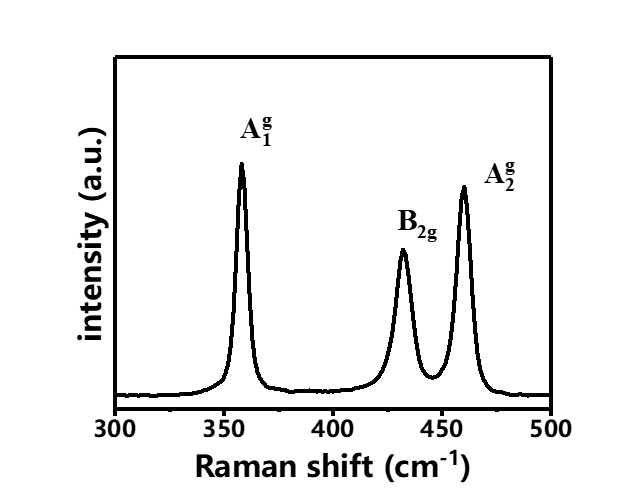


Figure S2. Raman scattering pattern of black phosphorus nanosheets.


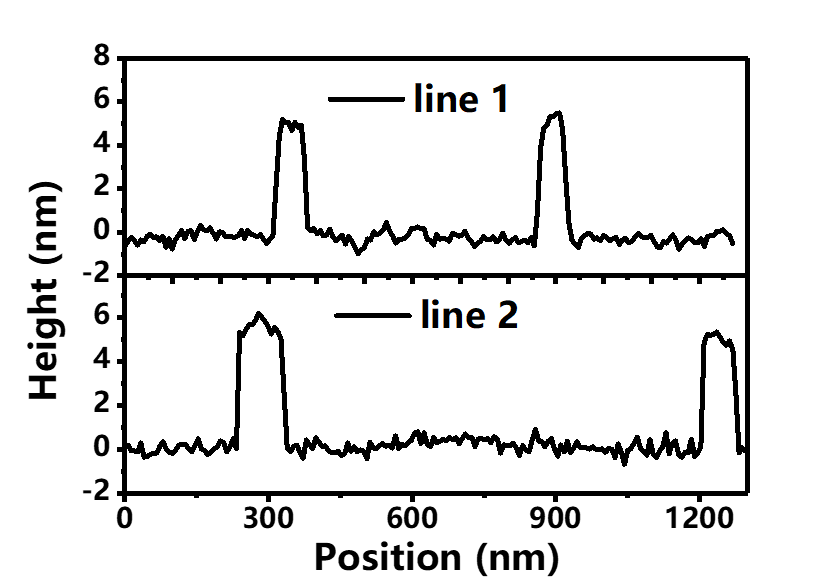

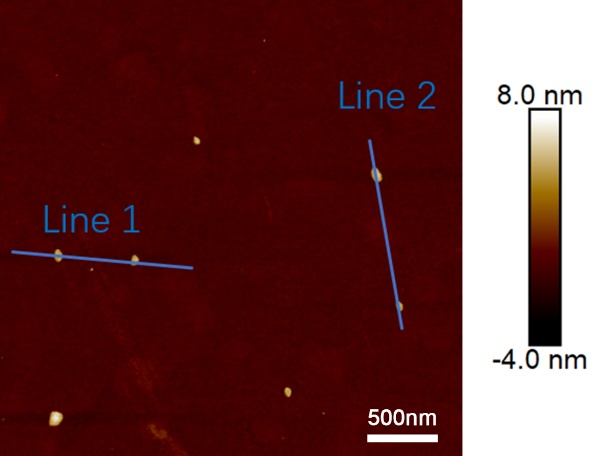


Figure S3. AFM image of black phosphorus nanosheets.


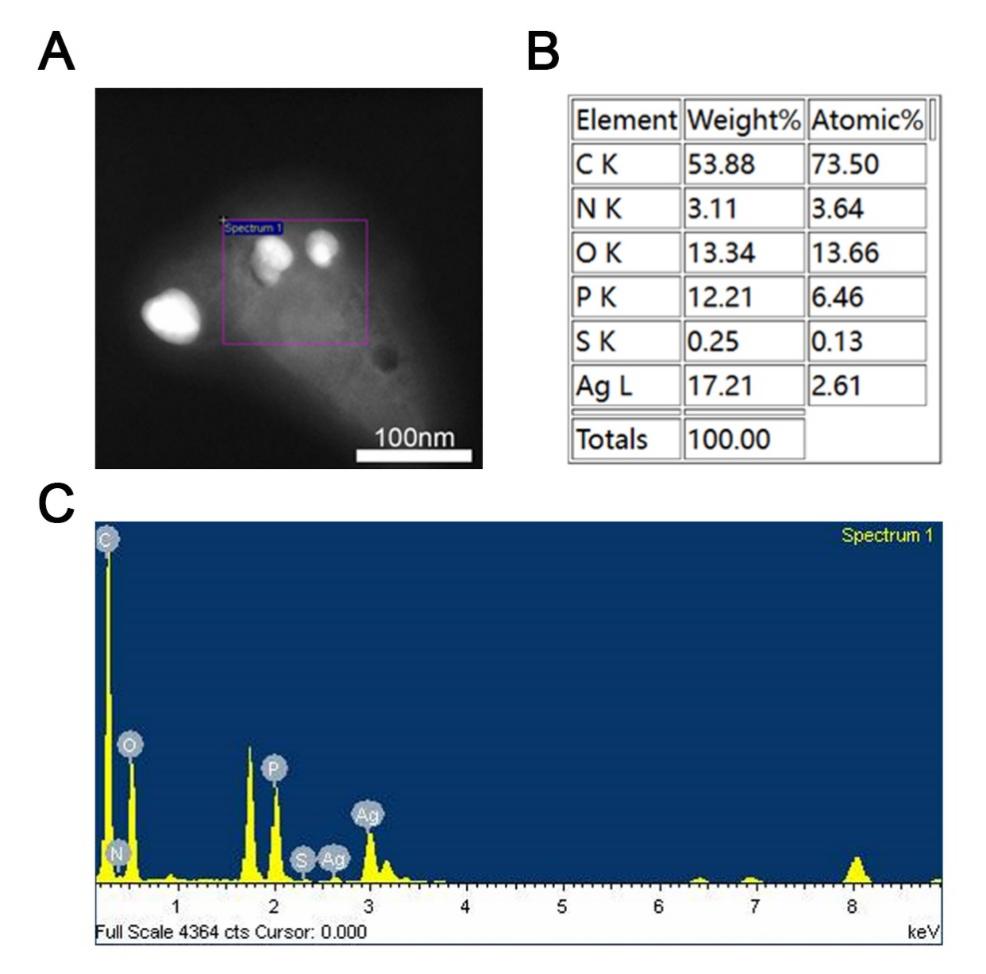


Figure S4. Mapping diagram of BP-PEG-AgSD. (A) TEM image of BP-PEG-AgSD. (B) Quantitative analysis chart of each element. (C) EDX image of TEM.


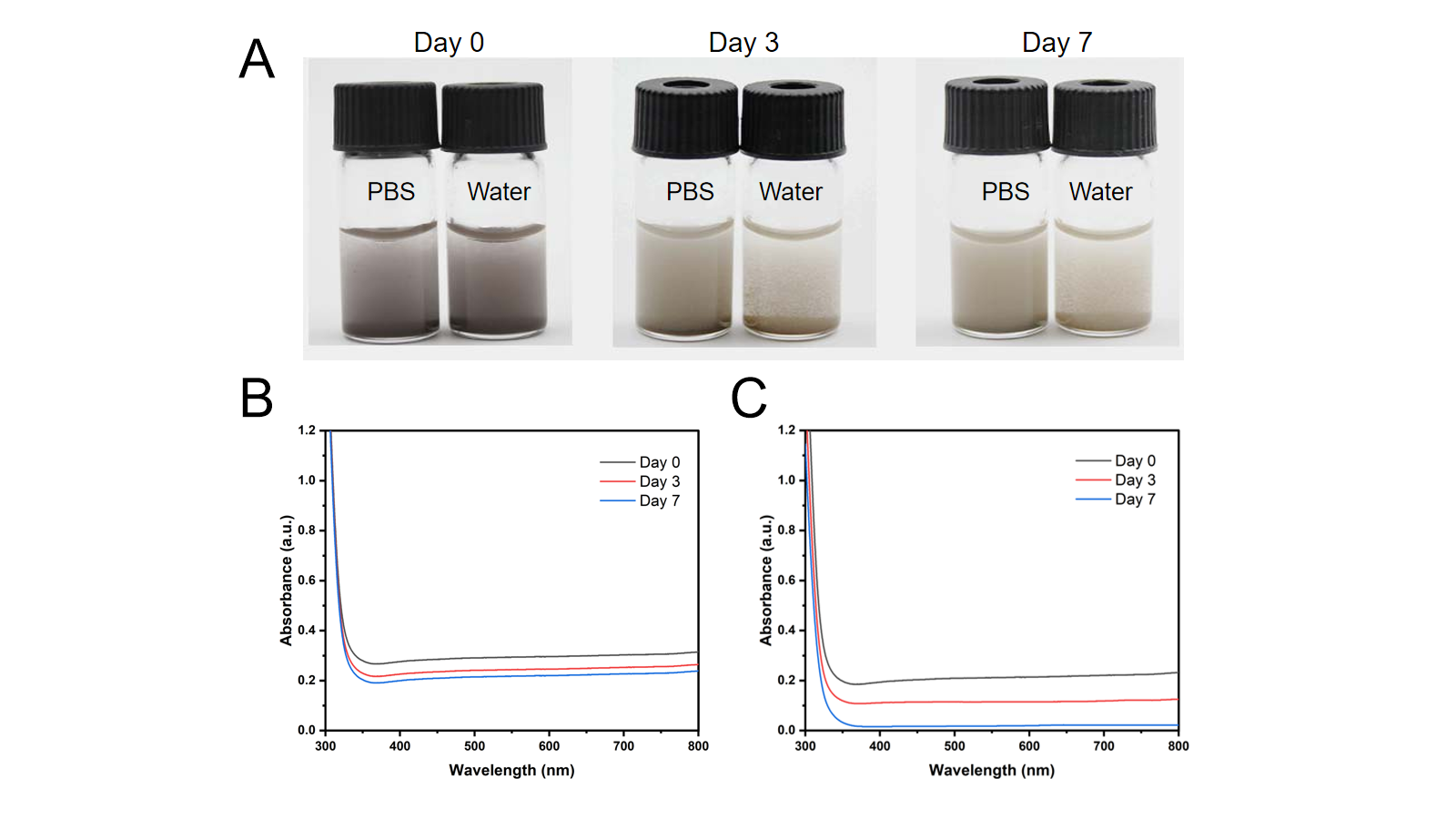


Figure S5. Stability performance of BP-PEG-AgSD solution. (A) Stability pictures of different time periods (B) and (C) Absorption spectra of BP-PEG-AgSD solution in PBS and water at day 0, day 3 and day 7.


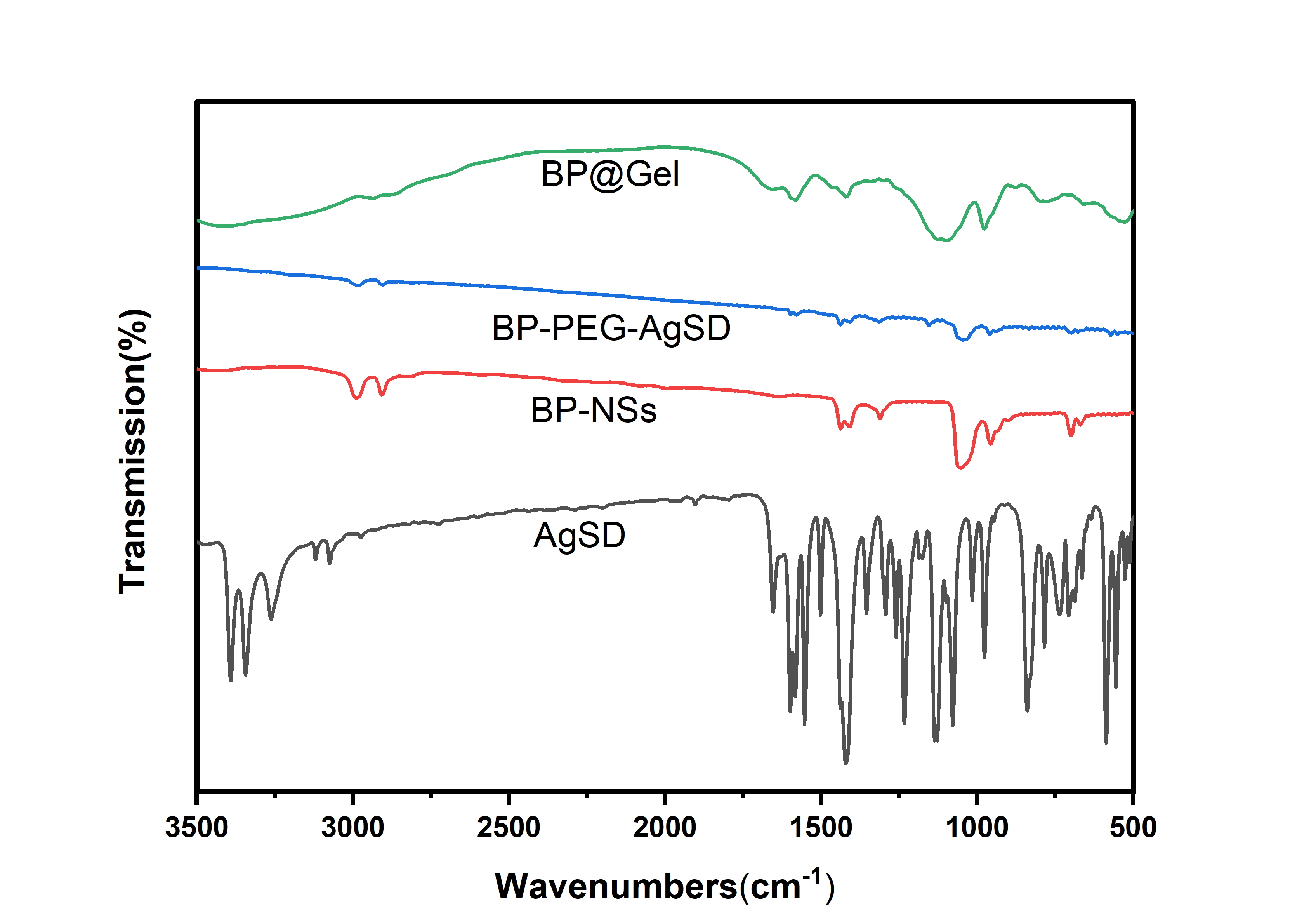


Figure S6.Infrared spectra of AgSD, BP-NSs, BP-PEG-AgSD and BP@Gel.


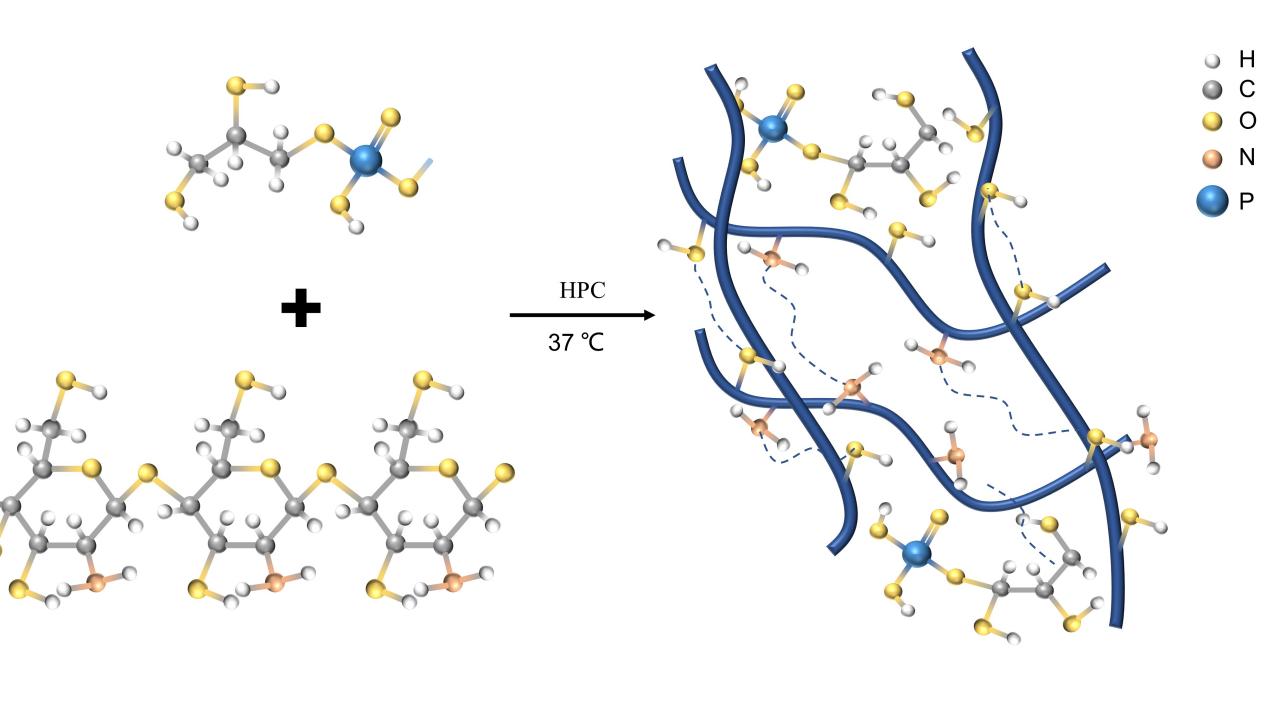


Figure S7. Reaction formula of chitosan, sodium *β*-glycerophosphate and hydroxypropyl cellulose.


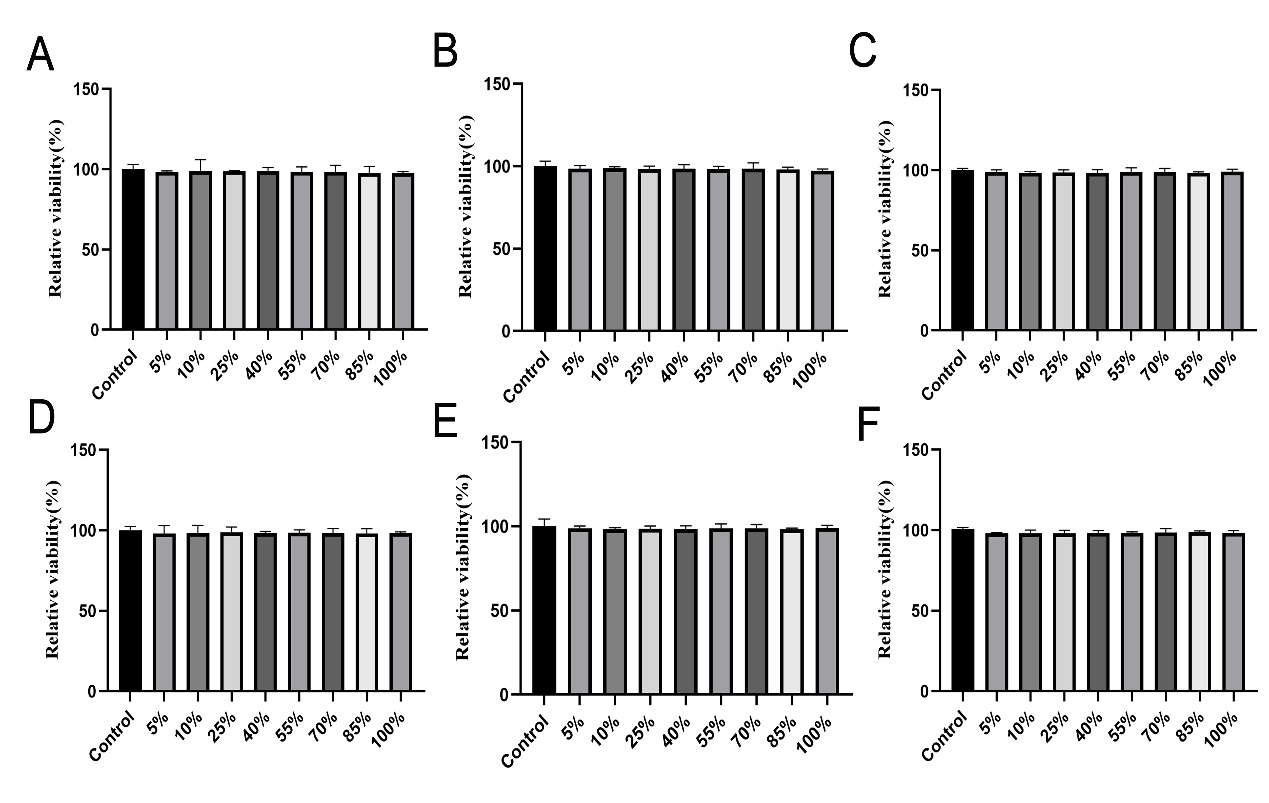


Figure S8. NIH3T3 cytotoxicity graph of Gel hydrogel group and BP@Gel hydrogel group. (A-C) Gel hydrogel groups at 24h, 48h and 72h. (D-F) BP@Gel hydrogel group at 24h, 48h and 72h.


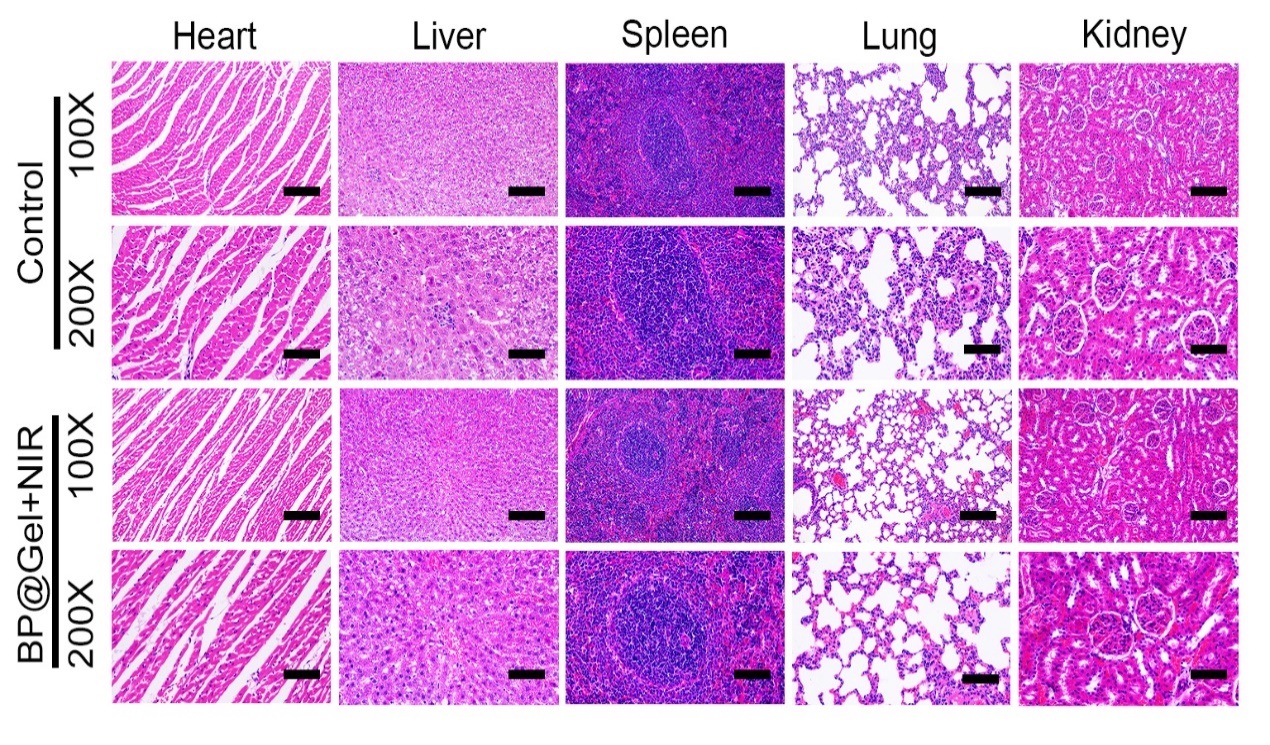


Figure S9. HE staining images of organs in Control group and BP@Gel hydrogel group. Scale bar: 100nm.
